# Objective quantification of the food proximity effect on grapes, chocolate and cracker consumption in a Swedish high school. A temporal analysis

**DOI:** 10.1371/journal.pone.0182172

**Published:** 2017-08-10

**Authors:** Billy Langlet, Petter Fagerberg, Andrew Glossner, Ioannis Ioakimidis

**Affiliations:** 1 Division of Applied Neuroendocrinology, Department of Neurobiology, Care Sciences and Society, Karolinska Institutet, Stockholm, Sweden; 2 Internationella Engelska Gymansiet Södermalm, Internationella Engelska Skolan, Stockholm, Sweden; University of Tennessee Health Science Center, UNITED STATES

## Abstract

Close food proximity leads to increased short-term energy intake, potentially contributing to the long-term development of obesity. However, its precise effects on eating behaviour are still unclear, especially with food available for extended periods of time. This study involved two similar high school student groups (15–17 years old), which had *ad libitum* access to grapes, chocolates and crackers during an hour-long experimental session. In the *distal* condition the foods were placed 6 meters away from the students (n = 24), in contrast to the *proximal* condition (n = 17) were the food was placed near the students. The identification of the type and the quantification of the amount of each food selected, for each individual serving, was facilitated through use of food scales and video recording. In the proximal condition individuals served themselves grapes and crackers more often and consumed more chocolate than in the distal condition. In total, participants in the proximal condition ingested significantly more energy (726 kcal vs. 504 kcal; p = 0.029), without reporting higher fullness. Food proximity also affected the temporal distribution of servings, with the first five minutes of the sessions corresponding to 53.1% and 45.6% of the total energy intake for the *distal* and *proximal* conditions, respectively. After the first five minutes, the servings in the *distal* condition were strongly clustered in time, with many students getting food together. In the *proximal* condition however, students displayed an unstructured pattern of servings over time. In conclusion, this study strengthens past evidence regarding the important role of food proximity on individual energy intake and, for the first time, it associates continuous food proximity to the emergence of unstructured eating over time. These conclusions, expanded upon by future studies, could support the creation of meaningful intervention strategies based on spatially and temporally controlled food availability.

## Introduction

Recent decades have seen a huge rise in obesity, with world prevalence having more than doubled in the adult population since 1980 [1]. The serious negative health effects of obesity are widely recognised [2], making obesity a significant financial burden for modern health care systems [3]. Weight gain occurs when energy intake is higher than energy expenditure [4], with small modifications in daily energy balance leading to significant weight changes over time [5]. High-energy foods, available at minimal physical effort, are certainly contributing to energy imbalance [6,7] and increased food availability is probably connected to both more frequent meal occurrences and increased meal size [8,9].

The determinants of food selection and consumption in naturalistic environments remain unclear [10,11], being difficult to quantify reliably, due to the predominant use of self-reporting [12] and the lack of quantitative data on eating behaviour patterns across a day [13]. However, recent research show positive correlations between availability of palatable foods on an individual's kitchen counter and their weight [14].

In controlled settings, short-term individual food intake can be modified by manipulating visual food cues [15], food packaging and plate sizes [16–18] and one’s dining companions [19,20]. Additionally, increased food availability is positively correlated with spontaneous eating [21] and increased meal sizes [22], even if the precise effects are again unclear [23]. Furthermore, both adults [24] and children [25] seem to eat more when food is available at a close distance.

Another potential behavioural factor affecting energy balance is the temporal distribution of eating over time, usually studied in the format of meal timing and frequency across a day [26]. The exact role of regularity on food intake and long term energy balance remains ambiguous, but there is some evidence that unstructured eating might contribute to the unbalancing of energy intake [27]. However, these studies often focus on the generalised effects of meal skipping or time-shifting [28] and it is often noted that novel methodologies for the collection of proper temporal data are required [29]. Similarly, most food proximity studies focus on group measures and only report cumulative effects at discrete time points, without accounting for temporal variations of individual eating behaviour [24,25,30], potentially ignoring the temporal effects of prolonged direct food availability on overall intake. Once more, for the temporal analysis of those effects on an individual level, more sophisticated measuring methodologies are required [28].

On the other hand, temporal analysis in single-meal individual eating behaviour, when quantified through the microstructural behavioural analysis [31] has proven valuable for the definition of individual eating styles associated with the development of obesity [32]. Similar methods have also been adopted very successfully in clinical settings for the treatment of eating disorders [33] and obesity [34]. Furthermore, past methodologies of temporal parallel analysis of weight-loss and video data used in the lab to fully describe single meals [35], have recently been adapted to the naturalistic analysis of adolescent eating behaviour during school lunches [36]. Similar techniques can now be reliably used to further analyse eating behaviour changes due to external food cue manipulations, such as food proximity.

By objectively quantifying human eating behaviour in a naturalistic setting, this study aimed at describing the underlying factors that drive human eating behaviour and can contribute to the development of obesity. The current study took place in a Swedish high school, where data was collected on the ingestive behaviour of students who were free to consume grapes, chocolate and crackers during a 60-minute work task. To achieve this, a previously developed technological tool was deployed (i.e., the Mandometer^®^ [33,34]), collecting continuous weight-loss data from common serving trays, with parallel video recordings, introducing a methodology for the combined analysis of serving sizes and serving occurrences across time on an individual level. Based on previous reports [24,25], we hypothesised that increased proximity to the food would result in increased energy intake over time per individual, expecting less structured temporal distribution of servings over time. The hypothesis was tested by comparing two groups of high school students of similar age and weight, who participated in testing sessions differing only in the spatial placement of the served food items.

## Material & methods

### Recruitment & subjects

Participants of the experiment were recruited from two first year natural science classes of a high school situated in central Stockholm. The recruitment took place in Feb 2015, as part of a bigger trial organised in the school through the EU project SPLENDID [37], with the same student sample participating in behaviourally monitored lunch sessions earlier during the test days [36]. A total of 41 out of 53 notified students agreed to participate in the experiment, providing written assent forms for themselves and written consent forms from their legal guardians. Randomisation among the two conditions was made between the two participating classes, resulting in 24 students assigned to the *distal* and 17 students to the *proximal* conditions (Table 1). Participation was non-discriminative, since every student was allowed to participate irrespective of their background, BMI or sex. Minimum sufficient sample sizes (n = 14) were calculated for a power of 0.80 for independent group comparisons, using expected effect size from a relevant report [25]. The presented protocol was approved by the Stockholm Regional Ethics Board and the presented practises fully follow the guidelines for human research in the Declaration of Helsinki [38].

**Table 1:** Group characteristics for the *distal* and *proximal* condition.

	*Distal (n = 24)*	*Proximal (n = 17)*
Female: Male (n)	13: 11	9: 8
Age (y)	16.6 (0.4)	16.8 (0.3) ^*ns*^
BMI (kg/m^2^)	20.9 (2.4)	21.7 (2.6) ^*ns*^

Data is presented as mean (SD), unless otherwise indicated. *ns*: p > 0.05.

### Procedure

#### Experimental preparation

**Fig 1:**
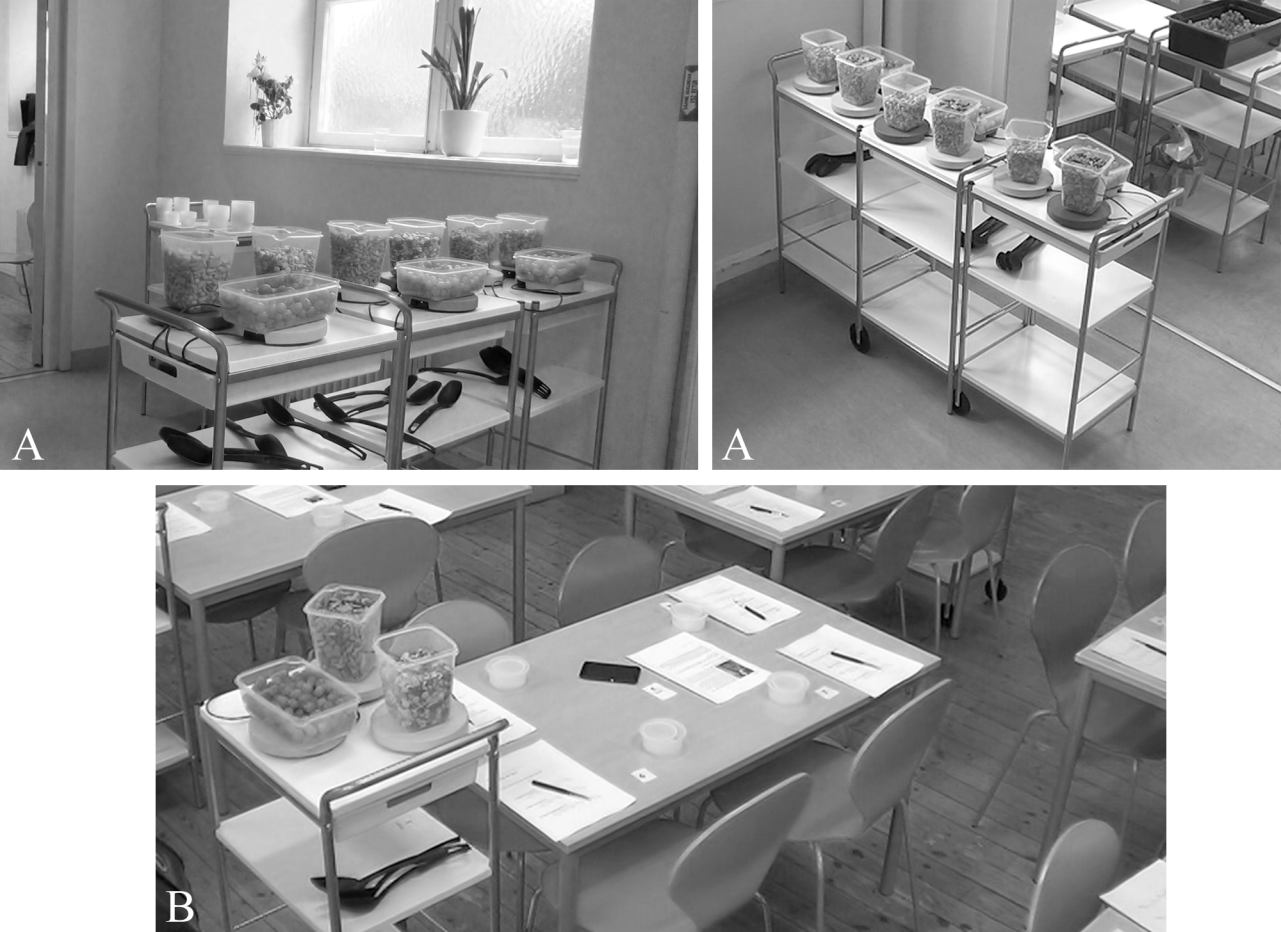
Experimental setup. The snack station positioning and the room in which the study was conducted, before receiving participants for the two tested conditions. A: *distal* and B: *proximal*.

The study followed a simple between-subject design. The two conditions were: i) *distal*, where food was situated at least 6 m away from the participants (Fig 1A) and ii) *proximal*, where food was situated at arm’s length from participants (Fig 1B). The two groups of students, each assigned to a single condition, were tested on separate school days, on two consecutive weeks. Two days prior to the testing the weight and height of the participants were measured by researchers. During the testing days, three hours before the initiation of the experimental sessions, the students participated in behaviourally monitored (but otherwise unhindered) school lunches with access to identical types of food, in a buffet setting, following the common school practises [36].

The two conditions were tested in the same room, which, for the duration of the trials, was only accessible by the researchers and the participants of the study and were video-recorded by 5 separate digital camcorders (Samsung, Suwon, South Korea) fully covering the room area. The required *snack stations* were prepared in advance, based on the needs of each condition.

#### Experimental sessions

The day of testing, after lunch, the participants followed their regular school schedule until 3:00 pm when they returned to the study area for the presented experiment. Upon arrival, the participants were freely (i.e., based on their personal preference, not in randomised fashion) divided into work groups of 3 or 4 students each and were placed at separate tables. At the beginning of the session (before given access to the food) the students answered an *appetite questionnaire*, containing questions about their perceived fullness, hunger and desire to eat. Each group was given a *work task* and students were informed that they could serve themselves food with no restrictions on the number of servings or serving sizes throughout the 60-minute work-sessions. Each student was provided with three small plastic serving cups of 100, 100 and 200 ml capacities for chocolate, crackers and grapes, respectively. The only restriction was that students were required to serve only themselves, in order to avoid the possibility of a single student carrying excess food for their whole workgroup. Food was removed at the end of the sessions and the leftover food items in each participant’s cup was weighed by researchers. The students then filled in the *appetite questionnaire* again, together with a *comfortability question* concerning their participation in the experimental session and the session was terminated.

#### Work task

The provided work task was identical for both testing sessions and it was not mentally or psychologically challenging, as it didn’t include any type of task performance measures and had no “failure” conditions. Specifically, the students were encouraged to freely interact with a smartphone, aiming at evaluating the functionality and the design of a novel app [39] and allowed the students freedom to socialise (inside their workgroup) and consume food throughout the session. The participants were also allowed to drink water ad libitum throughout the trials. While all the workgroups managed to finish the task before the end of the allocated 60 min period, the students were required to remain at their assigned positions until the end of the lecture period.

### Materials

#### Snack stations

Each station consisted of a kitchen-trolley equipped with three Mandometer^®^ scales (Fig 1A and 1B). Grapes, chocolate and crackers were served in separate, transparent plastic bowls. Each bowl was placed on a Mandometer^®^ scale, allowing separate weight measurements of each food type throughout the session. In all cases, the station bowls were fully refilled with food by researchers, when roughly one third of the bowl content was consumed in order to create a visual sensation of *ad libitum* food availability across the sessions.

In the *distal* condition, three *snack stations* were placed in a visible spot next to the main entrance of the classroom, at a distance of at least 6 meters from each workgroup desk (Fig 1A). In the *proximal* condition (Fig 1B) one *snack station* was placed adjacent to each workgroup desk (5 in total), within arm’s length of the participants.

#### Served food items

Three types of food items with differing macronutrient composition were provided (Table 2): i) green *grapes* (seedless, served without stalks), ii) *chocolate* lentils and iii) rice *crackers*. The nutritional value of chocolate and crackers were derived from the manufacturer’s labelling, while the nutritional value of grapes was derived from the database provided by Livsmedelsverket, the national food agency of Sweden (The National Food Agency food database, version 2016-02-17) [40].

**Table 2:** Macronutrient composition of the three provided food types.

	Grapes	Chocolate	Crackers
Carbohydrate (g/100g)	15.7	84.7	80.9
Fat (g/100g)	0.6	13.3	3.9
Protein (g/100g)	0.7	0.5	8.4
Total (kcal/100g)	75	460	366

All the served food types had a small unit size, allowing for precise quantification of individual intake. The types of food were selected in order to cover a wide range of energy densities and sensory characteristics in order to accommodate differing personal preferences of the participants.

#### The Mandometer^®^

The weight and the timing of each serving (i.e., each time a participant retrieved food from one of the food bowls) was recorded using the Mandometer^®^ v4 (Mikrodidakt, Lund, Sweden), a portable weighing scale linked to a small computer. The device records weight reduction over time at a sampling rate of 1Hz, providing raw weight data series in XML format.

#### Questionnaires

An *appetite questionnaire* was presented before and after the test session in order to estimate potential food intake after lunch and to evaluate appetitive differences between the groups. The intake between lunch and the experiment was answered in free text, while the remaining questions prompted participants to rate their hunger, fullness and desire to eat between 0-100mm on a Visual Analogue Scales (VAS), anchored by five-word descriptors; “Not at all”, “Little”, “Fairly”, “Very” and “Extremely” at 0, 25, 50, 75 and 100mm, respectively (see S1 File).

**Fig 2:**
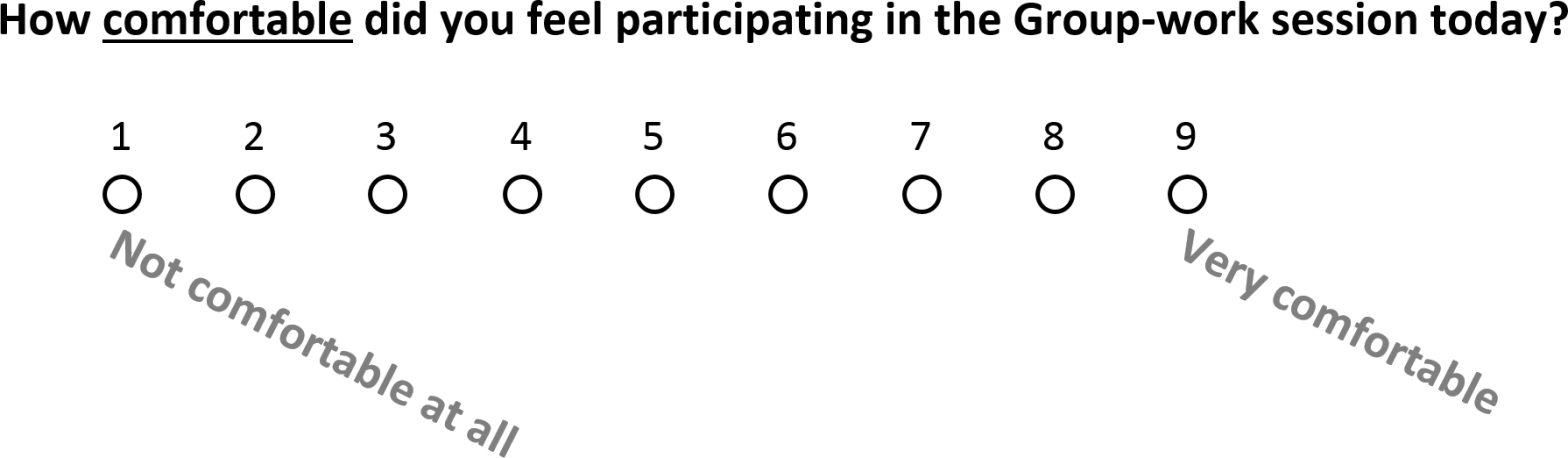
Likert scale. Comfortability question presented to participants on a Likert scale, after the experiment.

A *comfortability question* was used to evaluate perceived burden of the study, the participants rated their comfort on a 9-point Likert scale, with answers from 1: “not comfortable at all” to 9: “Very comfortable” (Fig 2).

The *appetite* and the *comfortability* questions were specifically constructed for the present study, based on the general principles of VAS [41] and Likert [42] scales. The *appetite questionnaire* was deployed for screening purposes only (i.e., to contextualise potential outlier values in the objectively recorded data) and was not one of the main outcome variables of the study. Similarly, the *comfortability* question was used to evaluate the acceptance of the research methodology by the target group of students. While not formally standardised, similar questions have been used in past research, both in controlled and naturalistic settings, in similarly-aged or older populations ([36,43]).

### Data handling

#### Data processing

The videos and Mandometer^®^ data were manually transferred to a PC after the sessions. The videos were annotated, using The Observer^®^ XT event log software (Noldus, Wageningen, the Netherlands). The time-stamp events were; *experiment start*, *experiment stop*, *grape serving*, *chocolate serving*, *cracker serving*, *social snack* (if a participant ate from another person's food) and *other* (used for unexpected behaviours, followed by a comment on the behaviour). During the experiment, no *social snack* or *other* event occurred. The Mandometer^®^ weight-loss data series were manually corrected, in order to remove recording artefacts (e.g., pressure put on the plate when handling the food, similar to [44]). Afterwards, the serving events from the video and weight-loss data from the Mandometer^®^ were matched and cross-validated, coupling each individual serving to a participant through the video. Finally, each participant’s weighed leftovers were subtracted from the quantified weight of the final respective serving.

The output of this analysis was a detailed, temporal description of the servings (weight and type of food) for each individual across the session. Note that this method enabled us to identify and measure the precise weight of each of the different food types at every visit to the *snack station*. Thus, the serving of each component is treated as a separate serving event, even if it occurred during the same visit to the *snack station*. If, for example, during one visit to the *snack station*, a participant served themselves 30g of food (consisting of 10g/10g/10g of grapes/chocolate/crackers, respectively), we report 3 different serving events of 10g each.

#### Statistical analysis

The presented statistical analyses and figures were done using Sigmaplot v13 (Systat Software Inc., San Jose, California) and R 3.2.3 [45]. The group characteristics and the questionnaire responses for the participating classes were compared with independent T-tests. Independent 2-way ANOVAs (post-hoc testing: Tukey’s) were used to compare: i) the energy intake of each food for individual participants across the testing conditions, ii) the average individual energy content per serving, across food types and testing conditions and iii) the number of servings for each individual across food types and testing conditions. Finally, the temporal distribution of servings across the testing conditions was tested with Kolmogorov-Smirnov test. The significance threshold of all statistical tests was set at 0.05 and all the presented values are mean (SD).

## Results

The characteristics of the students participating in the study were not significantly different between the two testing conditions (Table 1; p = 0.160 and p = 0.287 for age and BMI respectively).

### Cumulative energy intake per participant

Table 3 gives an overview of the mean energy intake of each food type per participant and the total energy intake per participant, in each condition (absolute food weights are presented in S1 Table).

**Table 3:** Energy intake per participant in the two experimental conditions.

	*Distal*	*Proximal*
Total intake (kcal)	503.5 (289.8)	726.2 (360.3)*
Grape intake (kcal)	170.2 (100.3)	176.4 (145.7)^*ns*^
Chocolate intake (kcal)	254.2 (241.9)	397.2 (262.2)*
Cracker intake (kcal)	79.1 (91.9)	152.6 (169.6)^*ns*^

Data is presented as mean (SD).

*: p < 0.05

*ns*: p > 0.05, 2-way-ANOVA, post-hoc testing.

**Fig 3:**
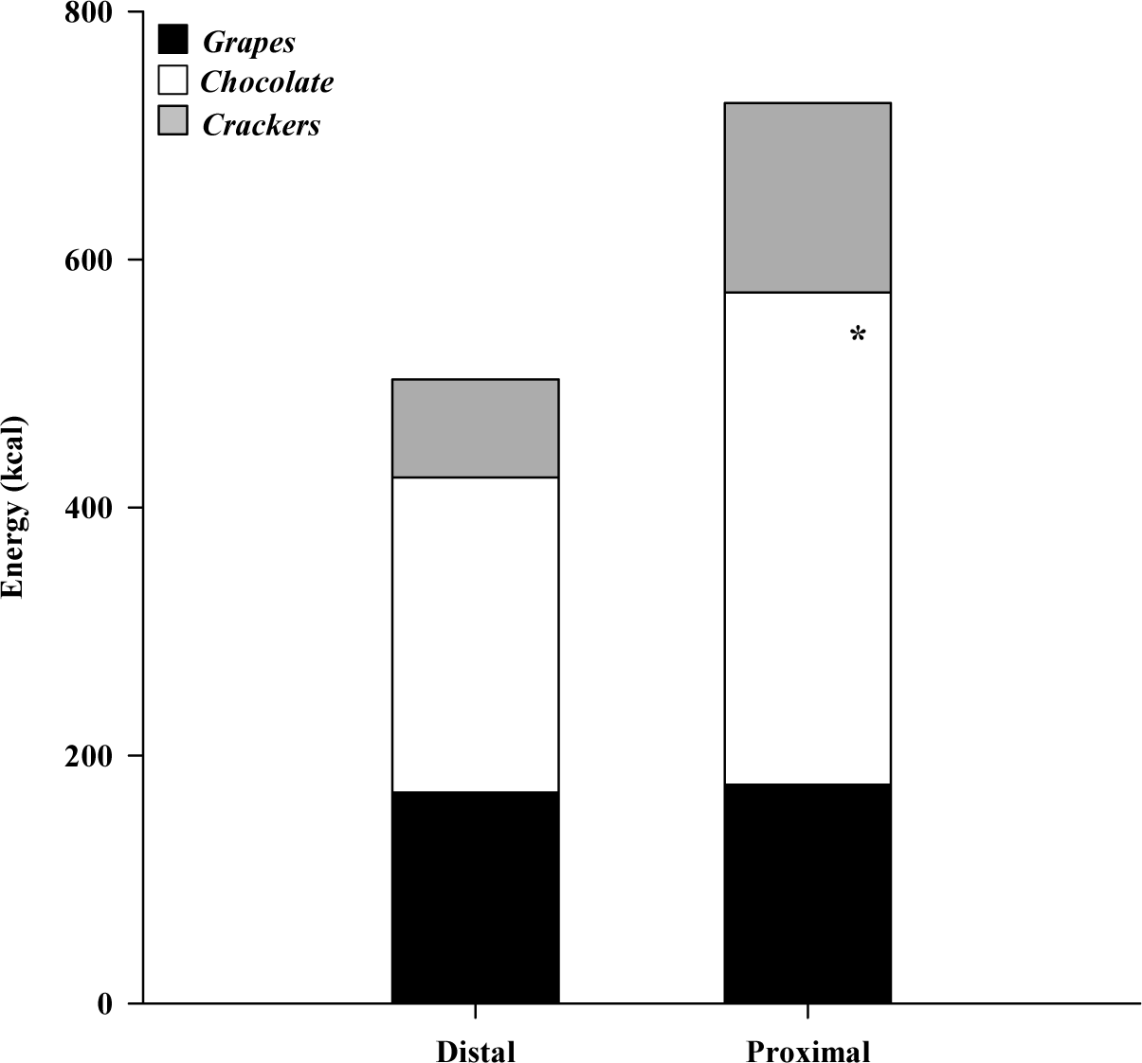
Mean energy intake per participant, per food type. Mean energy intake of grapes, chocolate and crackers per participant, after 60 minutes, in the distal and proximal condition, respectively. * p < 0.05 (post-hoc testing, 2-way ANOVA).

The comparison of the cumulative energy intake per participant, throughout the 60-min session, revealed a significant effect (p = 0.029) of the tested conditions (Table 3), with participants in the *proximal* condition ingesting, on average, 222.7 additional kcal (Fig 3). The post-hoc analysis (Table 3) between the two conditions for each of the consumed foods revealed no significant differences in the cumulative energy intake per individual due to grapes or crackers, (p = 0.913 and p = 0.246, respectively), but a significantly higher cumulative energy intake due to chocolate consumption in the *proximal* condition (p = 0.012).

### Serving characteristics per participant

Table 4 gives an overview of the differences across the two conditions in: i) the mean number of servings per food type, per individual, ii) the mean energy content of each serving, per food type, per individual (absolute food weights are presented in S2 Table).

**Table 4:** Serving event characteristics per participant in the two experimental conditions.

	*Distal*	*Proximal*
Number of servings (n)		
Grapes	1.9 (1.0)	3.3 (2.1)*
Chocolate	0.9 (0.8)	1.6 (1.1)^*ns*^
Crackers	1.1 (1.1)	2.4 (2.8)*
Energy content per serving (kcal)		
Grapes	80.5 (32.7)	53.1 (17.9)^*ns*^
Chocolate	187.0 (167.1)	260.0 (202.3)*
Crackers	43.5 (39.9)	58.9 (40.8)^*ns*^

Data is presented as mean (SD).

*: p < 0.05

and *ns*: p > 0.05, 2-way-ANOVA, post-hoc testing.

Overall, there was a significant effect of both *condition* (p < 0.001) and *food type* (p < 0.001) on the number of servings per individual (p < 0.001, condition effect). Post-hoc testing revealed that participants served themselves grapes and crackers more often in the *proximal* condition (p = 0.006 and p = 0.011, respectively), while there was no significant difference in individual chocolate serving numbers (p = 0.150). An interesting observation is that, while in the *distal* condition 38% (8 of 9 being women) of the participating students did not eat any chocolate, only 12% (2 of 2 being women) didn’t eat any chocolate in the *proximal* condition. The above observation *was not statistically tested* due to insufficient sample sizes.

**Fig 4:**
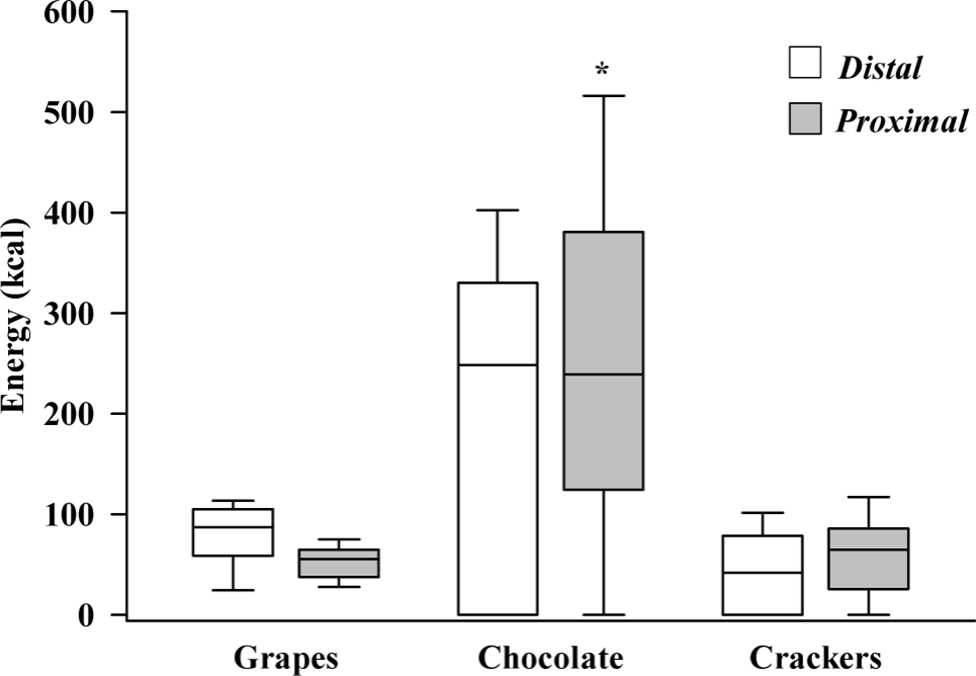
Total energy content (kcal) per serving per individual in the two tested conditions, for each food item. * p < 0.05 (post-hoc testing, 2-way ANOVA).

Furthermore, the participants served themselves more chocolate per serving in the *proximal* condition (p = 0.037), but there was no significant difference of energy content per serving of grapes and crackers (p = 0.430 and p = 0.656, respectively), as presented in Fig 4.

The presented values do not include the leftovers in the serving cups, which were 2.5 kcal, 33.2 kcal and 0.0 kcal in the *distal* condition and 1.1 kcal, 33.8 kcal and 2.2 kcal in the *proximal* condition for the grapes, chocolate and crackers, respectively (see leftover column in S1 Dataset).

In summary, in comparison to the *distal* condition, the participants in the *proximal* condition ingested foods of higher energy across the 60-minute sessions, when they served themselves grapes and crackers more often, while consuming larger amounts of chocolate per serving.

### Temporal analysis of servings

**Fig 5:**
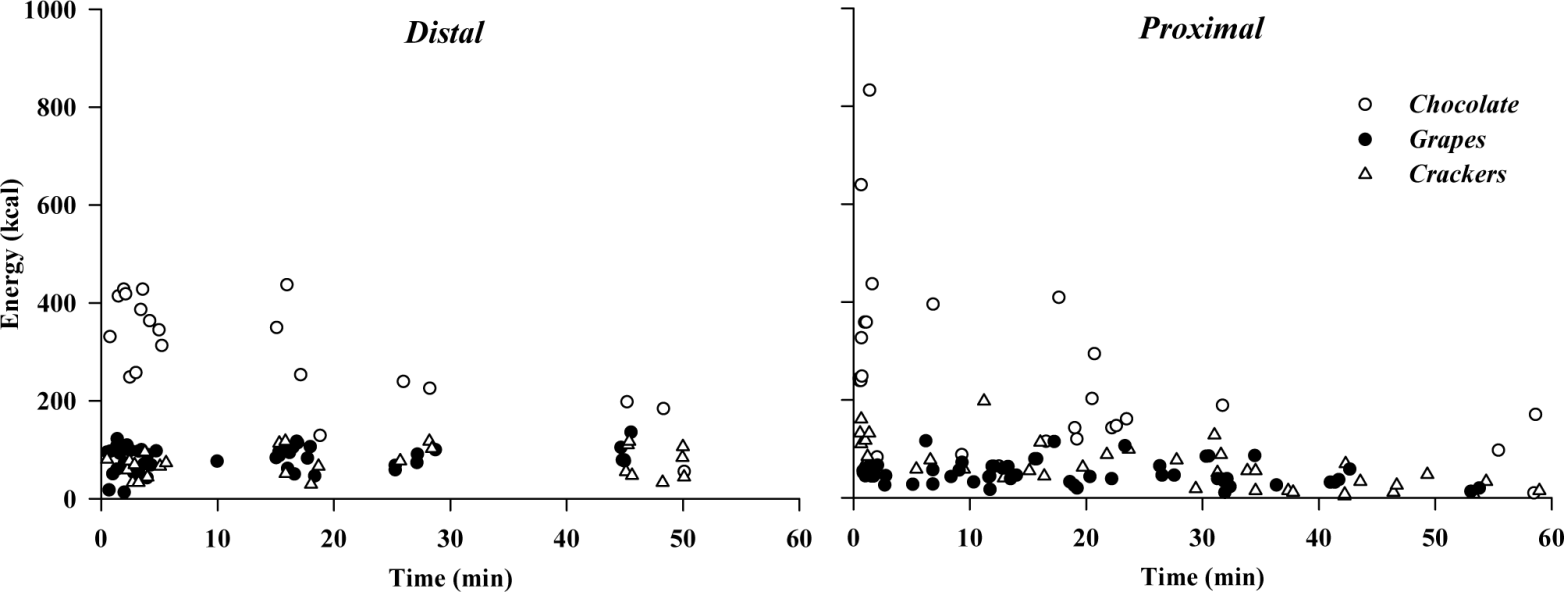
Temporal distribution of serving events. The time point and energy load for each serving event of grapes, chocolate and crackers, in each condition.

In both the *distal* and *proximal* conditions there was an initial burst of energy intake (Fig 5) with 53.1% and 45.6% of the total energy intake occurring in the first 5 minutes of each session, when 46.2% and 26.8% of the total number of servings occurred, respectively. The subsequent serving sizes across the session were reduced in both conditions, which also (Fig 5) differ in regard to the temporal grouping of the serving occurrences. Thus, after the first 5 min, in the *distal* condition servings appear grouped around specific time points, while in the *proximal* condition servings are spread across the session more evenly. A Kolmogorov–Smirnov test on the distribution of time differentials between subsequent servings (Δt = tx—t_x-1_, with t_x_: the time of the current serving and t_x-1_: the timing of the previous one), indicated that neither of the two conditions followed a normal distribution (p < 0.001), and also that there was a significant difference between conditions (p = 0.024). These results agree with the researchers’ observations at the time of the testing sessions, who perceived “continuous” servings during the *proximal* condition and “episodic” serving in the *distal* one.

### Subjective group measures

At the beginning of the experimental sessions, only two participants (one in each condition) reported any food intake after lunch. Their exclusion from the analysis did not modify the reported results, thus they are included in the presented analysis. Additionally, the two participating groups rated their perceived hunger, fullness and desire to eat similarly (p = 0.64, p = 0.82 and p = 0.55, respectively). Interestingly (Table 5), regardless of the energy intake differences, the two groups did not perceive any significant differences in any of these feelings at the end of the sessions either (p = 0.46, p = 0.97 and p = 0.69 for hunger, fullness and desire to eat, respectively).

**Table 5:** Subjective appetite measures in the two conditions.

	*Distal*	*Proximal*
Before experiment		
Hunger (0–100)	44.2 (20.9)	40.8 (24.4)^*ns*^
Fullness (0–100)	36.4 (15.3)	37.8 (25.4)^*ns*^
Desire to eat (0–100)	51.0 (19.9)	54.3 (26.7)^*ns*^
After experiment		
Hunger (0–100)	11.8 (13.2)	15.1 (14.6)^*ns*^
Fullness (0–100)	67.8 (21.7)	68.1 (24.2)^*ns*^
Desire to eat (0–100)	21.0 (22.0)	18.4 (18.4)^*ns*^

Data is presented as mean (SD). *ns*: p > 0.05, Independent t-tests.

At the end of the sessions, the participants in the two groups reported that they felt comfortable participating in the test sessions, with only one participant, across both groups, rating his/her comfort 5 out of 9 (on a Likert-scale), while 95% rated their comfort as 7 or above.

## Discussion

In the present study, we successfully tested the hypothesis that increased food proximity during a work task would lead to increased energy intake, through a differentiated pattern of servings across the testing sessions. In order to achieve that, we deployed a novel methodology in naturalistic conditions, based on the parallel analysis of weight-loss data from Mandometer^®^ and video extracted behavioural information, by adapting our own methodologies, previously used for single meal analysis in controlled [35] and naturalistic environments [36]. The *distal* and *proximal* conditions were identical, apart from the spatial placement of the available food items, and were tested in two independent samples of high school students of comparable BMI and age characteristics. The two independent student groups were also similar in regard to the quantity and the type of food which they consumed during the earlier lunch [36], while the majority of them (39 out of the 41 participants) did not report any food intake between lunch and the experimental sessions. Finally, the two groups had similar group ratings of perceived hunger, fullness and desire to eat at the beginning of the experimental sessions (Table 5) and the participating students felt comfortable taking part in the experiment, pointing towards high sample homogeneity in the performed group comparisons.

Considering the main outcomes of this study, the students participating in the *proximal* condition, ingested more energy over a period of 60 minutes (726kcal per individual) comparing to the students that participated in the *distal* condition (504kcal per individual). This was mainly caused by a significant increase of chocolate consumption in the *proximal* condition (397kcal vs 254kcal in the *distal* condition), while the cumulative energy intake for grapes and crackers was similar across the conditions (Table 3 and Fig 3). Interestingly, the significantly increased energy intake in the *proximal* condition did not result in significantly different subjective scores of fullness, hunger and desire to eat at the end of the session. Overall, these results support the notion that external food cues [15–20], and especially food availability [21,22] and food proximity have an important effect on the observed eating behaviour and the cumulative energy intake, at least short-term [24,25], potentially affecting energy balance throughout the day and, if continued over time, facilitate the development of obesity [5].

Looking closer into the characteristics of the average servings per individual across the testing sessions, we found that food proximity caused increased frequency of servings for both grapes and crackers (Table 4), which, however, was not enough to result in significant cumulative changes in energy intake for these food types. Also, the students with the food placed close to them during the scheduled work task ate more chocolate per serving (260kcal vs 187kcal per serving for the *proximal* vs the *distal* condition; Fig 4), resulting in the observed cumulative energy intake increase. In both conditions, almost half of the total energy intake was ingested in the first 5 minutes of the experimental session. However, there was a distinct difference in the temporal distribution of the subsequent servings between the conditions (Fig 5). Participants served themselves in regular intervals, or “bursts”, when the food was placed further away (i.e., *distal*), while they served themselves in an unstructured, “continuous” fashion when the foods were readily available (i.e., *proximal*). The “burst” serving behaviour in the *distal* condition was also noticed by the researchers on-site, with many participants visiting the *snack stations* around the same time, rather than by themselves.

The findings of the present study are mostly in line with previously reported results. For example, in a study conducted in an office setting, the difference of food unit consumption was quantified across a whole work day, when bowls of chocolate were placed either on the work desk (*proximal*), or 2m away (*distal*), resulting in 37.5% intake increase in the *proximal* condition [24]. However, the above study does not offer any objective information for the temporal distribution of the servings or individual serving characteristics, but expands upon the perceived “ease of access” of the *proximal* food.

Our findings also partially agree with previous reports examining the effects of food proximity on the consumption of foods of differing energy density [25,46]. In a previous study([46]) participants ingested significantly more from both types of food items (i.e., popcorns and apples) when they were placed in close proximity, albeit for a limited time period (6 min), while in the study by Musher-Eizenman [25], again, both the high- and low- energy food items were consumed more when proximal, but the duration of food availability is not reported. This difference in the duration of the availability of the food might be responsible for the discrepancy in our results, since in the presented study, the total energy intake does not differ for the two foods of lower energy density (grapes and crackers). Overall, our study supports the notion that unstructured eating, in this case facilitated by continuous food availability due to increased proximity, can lead to increased energy intake over time [28]. Past studies, however, are not directly comparable to the presented work, as they are based on subjective measures of food intake [29] and they have mainly focused on irregular daily meal occurrence [26], rather than controlled efforts to describe the temporal pattern of eating.

As stated before [30], one should be careful regarding the limitations of short experimental protocols (e.g., 6 min of food availability in [46]), avoiding generalisation of the results for longer time periods. This also points toward the importance of continuous data collection for understanding food selection and ingestion in certain settings, with our study being the first, to our knowledge, providing detailed reports on these measures. On the other hand, that does not reduce the usefulness of time-restricted study protocols, which might be better suited for modelling eating behaviour in time-restrained environments (e.g., a school meal/class break under strict school schedule [47]).

The lack of additional perceived fullness after the increased energy intake in the *proximal* condition in our study, supports our previously stated opinion [48] about the disconnection between the objectively quantified behaviour and perceived subjective measures, at least in conditions when “mindless” [49] or “automatic” [50] eating is facilitated. These findings should further discourage the use of subjective measures (e.g, satiety, hunger and fullness) as sole predictors or descriptors of energy intake in research, in agreement with past suggestions [43,51,52].

The greatest strength of the present study is that, in contrast to similar studies examining the effects of various external factors on eating [15,17,18,20,24,25], the deployed methods allowed for detailed continuous recording of servings per participant over time, which enabled us to compare the time distribution of servings and energy intake between the two conditions. The identical nature of every aspect of the protocol and the sample homogeneity across the two conditions are also important, allowing for proper comparisons between the independent groups.

A factor which can potentially be regarded as a weakness of our protocol is the specific setting of the study. In the everyday school practice students do not usually have access to food items during formal work tasks, making the setting of the study mostly relevant to non-supervised school work, usually taking place later in the school day, at least for the selected target group. The sample size in this study is also relatively small, and while the power of the presented comparisons was adequate, we could not test some potentially interesting differences between conditions, such as the observed tendency of females to avoid chocolate altogether in the *distal* but not in the *proximal* condition.

Thus, future controlled and semi-controlled research should aim to quantify in detail additional parameters which might interact with the presented effects, focusing on sex (male vs female), population (e.g., obese vs normal weight), or setting (e.g., office-scape, family environment, etc.) differences. On the other hand, in naturalistic settings, the logical next step is the deployment of comparable measuring techniques over extended periods of time, in order to gather further quantitative information on the distribution of eating occurrences, together with more exact data on the energy loads of individual food items. Such an achievement would close the gaps of knowledge concerning the contribution of different eating occurrences on obesity development [10,11], remedying the identified inconsistencies in self-reported energy intake [12]. To that effect, new technological advancements are required (e.g., note the effort in current research projects like SPLENDID [53] and BigO [54]) and one can envision a comparable system, where the video recordings are replaced by automatic food item registration (e.g., QR code scanning [55] etc.). Additional advancements in data analysis (e.g., [56] or [57]) can facilitate automatic extraction of the recorded information, facilitating a “hands-off” (but not less detailed) approach to the analysis of the eating behaviour in different populations. In the future that can lead towards the deployment of more meaningful intervention and prevention schemes, carefully controlling the timing and the proximity of food servings, in order to limit unstructured occurrences of eating.

## Conclusions

In the present study, food proximity, especially of chocolate, appears to be responsible for increased cumulative energy intake per participant when two independent groups of high school students were tested in identical naturalistic conditions. Additionally, the proximity of the food seems to affect the pattern of servings across time, causing increased frequency of servings for grapes and crackers and, in general, leading to “continuous” rather than “burst” serving patterns. Overall, these findings provide a more comprehensive description of the reported effects of food proximity on energy intake, compared to past studies [24,25]. They also indicate the importance of controlling the accessibility of food in studies examining other external factors, such as social interactions (e.g., [20,58]), in respect to their effect on food intake. In the future, detailed temporal analysis might prove a powerful tool to analyse eating behaviour in naturalistic environments with continuous access to food for longer periods of time (e.g., an office environment [24], a kitchen environment [14], etc.), allowing the collection of more detailed information about the environmental effects on eating behaviour.

## Supporting information

S1 Dataset Subject measures. Data on sex, age, BMI, questionnaire answers and food item leftovers, for each participant.
(XLSX)

S2 Dataset Event log of experiment. Data on the size and time of each serving, for each participant.
(XLSX)

S1 File Before and after meal questionnaire. The before and after meal questions used in the study as presented to the participants.
(PDF)

S1 Table Food weight ingested per participant. (DOCX)

S2 Table Food weight per serving. (DOCX)
