# Supplementary material for: Objective quantification of the food proximity effect on grapes, chocolate and cracker consumption in a Swedish high school. A temporal analysis
Source: PLoS One. 2017 Aug 10;12(8):e0182172. doi: 10.1371/journal.pone.0182172 (PMC5552216; doi:10.1371/journal.pone.0182172)
Supplement: S1 File — The before and after meal questions used in the study as presented to the participants. (PDF) [file pone.0182172.s003.pdf]

Name:………………………………….

To be filled in ***<u>before</u>*** *the session*

Food intake since lunch

Describe in detail what you have eaten after lunch until now:

……………………………………………………………………………………………

……………………………………………………………………………………………

……………………………………………………………………………………………

How <u>hungry</u> do you feel right now?

Not at all | Little | Fairly | Very | Extremely

How <u>full</u> do you feel right now?

Not at all | Little | Fairly | Very | Extremely

How strong is your <u>desire to eat</u> right now?

Not at all | Little | Fairly | Very | Extremely

*To be filled in **after** the session*

How hungry do you feel right now?

Not at all — Little — Fairly — Very — Extremely

How full do you feel right now?

Not at all — Little — Fairly — Very — Extremely

How strong is your desire to eat right now?

Not at all — Little — Fairly — Very — Extremely
