# Supplementary material for: Objective quantification of the food proximity effect on grapes, chocolate and cracker consumption in a Swedish high school. A temporal analysis
Source: PLoS One. 2017 Aug 10;12(8):e0182172. doi: 10.1371/journal.pone.0182172 (PMC5552216; doi:10.1371/journal.pone.0182172)
Supplement: S1 Table — (DOCX) [file pone.0182172.s004.docx]

S1 Table. Absolute food weight (g) ingested per participant in the *distal* and *proximal* conditions.

|  | ***Distal*** | ***Proximal*** |
| --- | --- | --- |
| Food intake (g) |  |  |
| Total | 303.8 (149.9) | 363.2 (226.2) |
| Grapes | 227.0 (133.8) | 235.2 (194.3) |
| Chocolate | 55.3 (52.6) | 86.4 (57.0) |
| Crackers | 21.6 (25.1) | 41.7 (46.3) |

Data is presented as mean (SD).
