# Supplementary material for: Objective quantification of the food proximity effect on grapes, chocolate and cracker consumption in a Swedish high school. A temporal analysis
Source: PLoS One. 2017 Aug 10;12(8):e0182172. doi: 10.1371/journal.pone.0182172 (PMC5552216; doi:10.1371/journal.pone.0182172)
Supplement: S2 Table — (DOCX) [file pone.0182172.s005.docx]

S2 Table. Absolute weight per serving, per food type, per participant in the two experimental conditions.

|  | ***Distal*** | ***Proximal*** |
| --- | --- | --- |
| Weight per serving (g) | | |
| Grapes | 107.3 (43.5) | 70.8 (23.8) |
| Chocolate | 40.6 (36.3) | 56.5 (44.0) |
| Crackers | 11.9 (10.9) | 16.1 (11.2) |

Data is presented as mean (SD).
